# Asymmetrical and smaller size of trunk muscles in combat sports athletes with lumbar intervertebral disc degeneration

**DOI:** 10.1186/s40064-016-3155-8

**Published:** 2016-09-01

**Authors:** Kazunori Iwai, Koji Koyama, Takashi Okada, Koichi Nakazato, Ray Takahashi, Shingo Matsumoto, Yosuke Yamamoto, Kenji Hiranuma

**Affiliations:** 1 School of Kinesiology, Western University, 1151 Richmond Street, London, ON N6A 3K7 Canada; 2 Hiroshima Maritime College, National Institute of Technology, 4272-1 Higashino Osakikamijima-cho, Toyota-gun, Hiroshima, 725-0231 Japan; 3 Department of Judotherapy, Tokyo Ariake University of Medical and Health Science, 2-9-1 Ariake, Koto-ku, Tokyo, 135-0063 Japan; 4 Faculty of Sport Science, Nippon Sport Science University, 7-1-1 Fukasawa, Setagaya-ku, Tokyo, 158-8508 Japan; 5 Graduate School of Health and Sport Science, Nippon Sport Science University, 7-1-1 Fukasawa, Setagaya-ku, Tokyo, 158-8508 Japan; 6 Sports Methodology (Wrestling), Nippon Sport Science University, 1221-1, Kamoshida-cho, Aoba-ku, Yokohama, Kanagawa 227-0033 Japan; 7 Sports Methodology (Judo), Nippon Sport Science University, 7-1-1 Fukasawa, Setagaya-ku, Tokyo, 158-8508 Japan

**Keywords:** Radiological abnormality, Asymmetrical anatomy, Muscle atrophy, Contact sports

## Abstract

**Background:**

Lumbar intervertebral disc degeneration (LDD) frequently occurs in athletes. Associations between LDD and trunk muscles still remain unclear.

**Purpose:**

This study examined whether there is an association between the prevalence of LDD and the symmetry and size of the cross-sectional areas (CSAs) of the trunk muscles in combat sports athletes.

**Methods:**

Participants in this study were 151 collegiate male combat sports athletes. A total of 755 lumbar intervertebral discs from L1–2 to L5–S1 in 151 athletes were assessed using magnetic resonance imaging (MRI) and a comprehensive grading system of LDD (grades I–V). All 151 athletes were divided into 2 groups: LDD and non-LDD. CSAs of trunk muscles at the L3–4 disc level were measured using MRI.

**Results:**

Sixty-nine athletes had LDD at 1 or more disc levels (45.7 %). The LDD grade for the lower 2 disc levels was significantly higher than that for the other disc levels (*p* < 0.001). The CSAs of the left and right sides in trunk muscles were significantly asymmetrical, independent of the LDD which was prevalent in the disc levels (obliques: *p* = 0.040; quadratus lumborum: *p* < 0.001). The relative CSAs of trunk muscles to their body weight in the LDD group were significantly smaller than those in the non-LDD group (rectus abdominis: *p* = 0.011; obliques: *p* = 0.024; quadratus lumborum: *p* = 0.006; lumbar erector spinae plus multifidus: *p* = 0.001).

**Conclusion:**

This study suggests that the prevalence of LDD is associated with asymmetrical and relatively smaller CSAs of trunk muscles in combat sports athletes.

## Background

Lumbar intervertebral disc degeneration (LDD) is one of the most common structural abnormalities in the lumbar region of the spine. Risk factors for LDD include aging, body weight, sports activities, and genetic inheritance in human beings (Ala-Kokko 2002; Battie et al. 2004; Bono 2004; Clark et al. 2009; Elfering et al. 2002; Liuke et al. 2005; Parkkola and Kormano 1992). LDD is characterized and graded radiologically by the presence of an inhomogeneous structural disc showing gray or black discoloration, an unclear or absent distinction between the nucleus and annulus, and disc height reduction in T2-weighted images on magnetic resonance imaging (MRI) (Pfirrmann et al. 2001). A large number of the reviews demonstrated that Pfirrmann’s grading system with LDD was frequently used in the previous scientific studies (Koyama et al. 2015; Kulling et al. 2014; Min et al. 2010; Salamat et al. 2016).

LDD frequently occurs in various sports played at the elite and intercollegiate levels (Baranto et al. 2009; Hangai et al. 2009; Hellstrom et al. 1990; Koyama et al. 2013; Kulling et al. 2014; Ong et al. 2003). Hangai et al. (2009) showed that 26–60 % of competitive athletes playing 6 different types of sports had LDD in at least 1 lumbar disc level between L1–2 and L5–S1. Moreover, a high incidence and prevalence of LDD (56–100 %) was found in sports with high demands on the spine, such as combat sports (wrestling and judo) and weight lifting (Baranto et al. 2009; Hellstrom et al. 1990; Okada et al. 2007).

Trunk muscles have variable sizes, shapes, and principally function to stabilize, flex, extend, and rotate the lumbar spine (Iwai et al. 2008; Ranson et al. 2008). Trunk muscles are roughly comprised of the following 5 types: rectus abdominis, obliques, psoas, quadratus lumborum, and lumbar erector spinae plus multifidus (Iwai et al. 2008; Kubo et al. 2007). Several papers reported that cross-sectional areas (CSAs) of the trunk muscles have been associated with low back pain (LBP, Demoulin et al. 2007; Keller et al. 2004). CSAs of the trunk muscles in individuals with LBP show 2 types of morphological changes: differences in the asymmetry of left and right sided trunk muscles and decreases in their size.

Previous studies have described that individuals with acute and chronic LBP have significantly smaller CSAs of trunk muscles than those without (Clark et al. 2009; Danneels et al. 2000; Demoulin et al. 2007). Danneels et al. (2000) found that CSAs of paravertebral muscles, including multifidus, in patients with chronic LBP were smaller than those in healthy volunteers. The asymmetrical CSAs of trunk muscles have also been associated with the prevalence of LBP. Barker et al. (2004) demonstrated a significant asymmetry of the psoas and multifidus muscles between the left and right sides in patients with unilateral LBP. Clark et al. (2009) reported that quadratus lumborum showed a significantly greater asymmetry between the right and left sides in an acute LBP group compared to that in a control group.

Asymmetrical CSAs of the trunk muscles, particularly the lumbar multifidus muscle, have been reportedly associated not only with acute and chronic LBP, but also with some intervertebral disc and nerve lesions (e.g. lumbosacral radiculopathy, Altinkaya and Cekinmez 2016; Hodges et al. 2006; Hyun et al. 2007; Kulig et al. 2009). Asymmetry and decreased size of the multifidus muscle have been found to be adjacent to the disc in patients who had unilateral lumbosacral radiculopathy with a herniated intervertebral disc and were scheduled for single-level lumbar microdiscectomy (Hyun et al. 2007; Kulig et al. 2009). In addition, the multifidus muscle rapidly decreased in size after the occurrence of lumbar intervertebral disc lesions in an animal study (Hodges et al. 2006). These studies were mainly conducted to assess CSAs of psoas, lumbar erector spinae and multifidus muscle. However, little is known about the overall association between LDD and CSAs of the other trunk muscles.

The purpose of this study was to investigate the association between the symmetry and size of the trunk muscle CSAs and the prevalence of LDD in combat sports athletes. We hypothesized that there are significant associations between the asymmetry for the left and right sides of the trunk muscles and the prevalence of LDD. It was also hypothesized that the relative size of trunk muscles to their body weight in combat sports athletes with LDD are significantly different than those without LDD group. This association could appear more evident among those combat sports athletes who incur greater loads on the lumbar region (Iwai et al. 2004; Okada et al. 2007).

## Methods

### Experimental design

To confirm our hypotheses, CSAs of the trunk muscles and physical characteristics were measured by using various devices. Also, LDD was assessed in the way hereinafter prescribed. We compared the asymmetry between left and right sides and size of trunk muscles in collegiate male combat sports athletes with and without LDD.

### Participants

A group of 151 collegiate male combat sports athletes, including 50 wrestlers and 101 judokas, participated in this study. The combat sports athletes were selected from the trained athletes attending the Nippon Sport Science University in Japan. All of the participants were Japanese collegiate high-level athletes who volunteered for the study. All athletes regularly spent approximately 4 h per day (2 times a day, 6 days a week) practicing their combat sports. The purpose of this study and protocol were explained to all athletes and their coaches, and signed informed consent was obtained prior to their participation. The study was approved by the Ethical Committee of the university.

### Physical characteristics

Anthropometric data of the athletes were recorded (height to the nearest 0.1 cm and body weight to the nearest 0.1 kg). Body mass index (BMI) was calculated as body weight in kilograms divided by height in square meters (kg/m^2^). Moreover, the age and combat sports experience of each athlete were investigated.

### Assessment of LDD

The athletes lay on a bed in the MR imaging unit in a comfortable and relaxed supine position. MR imaging was performed with a 0.3-T MR using surface coils in the supine position (AIRIS II, Hitachi, Tokyo, Japan). T2-weighted fast spin-echo imaging was used to obtain sagittal images of the lumbar spine and intervertebral discs (repetition time, 3000 ms; echo time, 112 ms; matrix, 256 × 265; field of view, 320 mm; slice thickness, 10 mm).

All MR images, taken at 5 lumbar intervertebral disc levels from the first lumbar (L1) vertebra to the first sacral vertebra (S1), were independently evaluated by 2 experienced orthopedic specialists in a random order using a grading system for LDD assessment. Using a comprehensive grading system for LDD, discs were classified into 5 grades, as described by Pfirrmann et al. (2001). This system uses characteristics of disc structure, distinction between the nucleus and annulus, MRI signal intensity, and intervertebral disc height for grading. This comprehensive grading system for LDD has been accepted as a standard (Pfirrmann et al. 2001) and reliable evaluation tool for assessment of MRI disc morphology (Hangai et al. 2009; Koyama et al. 2015; Kulling et al. 2014; Min et al. 2010; Salamat et al. 2016). The assessment was blinded so as not to disclose any knowledge about the athlete’s conditions. When the 2 experienced orthopedic specialists had differing opinions on disc grades, the disagreements were debated and discussed until a resolution was reached.

The 151 participating athletes were divided into 2 groups: LDD and non-LDD. The LDD group included participants with at least 1 abnormal disc from L1–2 to L5–S1 of grade III, IV, or V. The non-LDD group included subjects with 5 normal discs of grade I or II.

### CSAs of trunk muscles

**Fig. 1:**
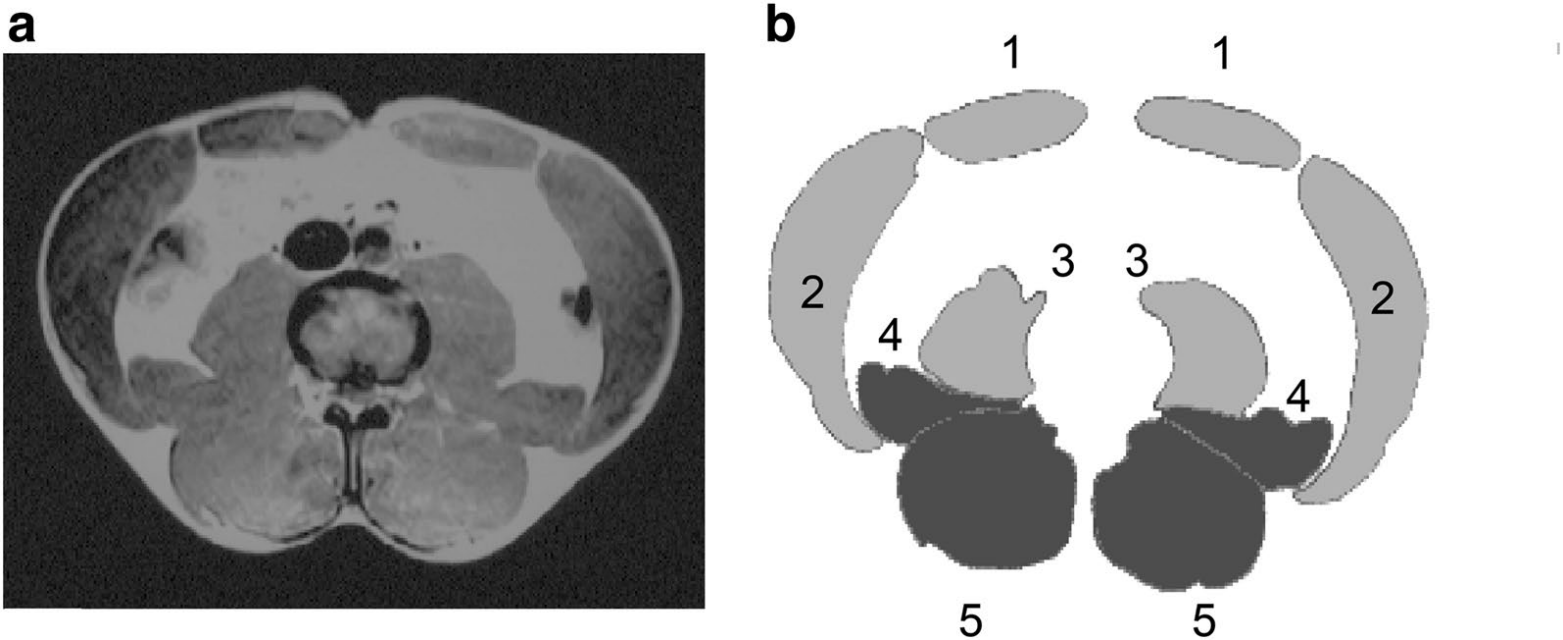
Transverse (**a**) and traced (**b**) images of the trunk muscles at the L3–4 disc level. In the present study, the cross-sectional areas (CSAs) on each side of midline are grouped in 5 large areas (*1* rectus abdominis, *2* obliques, *3* psoas, *4* quadratus lumborum, *5* lumbar erector spinae plus multifidus)

Transverse MR spin-echo T1-weighted images were obtained at the L3–4 level parallel to the lumbar disc space in order to minimize inter-participant differences in anatomical curvature of the lumbar spine (Fig. 1a, repetition time, 760 ms; echo time, 20 ms; matrix, 256 × 265; field of view, 320 mm; slice thickness, 5.0 mm). The image was traced onto paper and the traced image was then transferred to a computer in order to measure CSAs (Shown in Fig. 1b). CSAs were calculated using image analysis software (Scion Image Beta 4.02, Scion Corp., Frederick, MD, USA), and grouped into 5 large areas because the individual muscles had poorly defined borders. Each of the 5 areas was represented by the sum of the CSA on the left and right sides of the transverse image (rectus abdominis, obliques, psoas, quadratus lumborum, and lumbar erector spinae plus multifidus). The 5 areas were summed to obtain the total area. Three of the CSAs included multiple muscles: obliques, psoas, and lumbar erector spinae). Oblique muscles comprise the internal and external obliques and transversus abdominis. Psoas muscles comprise the psoas major and minor muscles. The lumbar erector spinae comprises the iliocostalis, longissimus, and spinalis. All CSAs were also normalized by dividing the values by the athlete’s body weight. This method was used in the previous study (Peltonen et al. 1998), in order to indirectly eliminate differences in their lean body mass.

### Data analysis

All statistical analyses were evaluated using PASW Statistics 18 software (SPSS Japan Inc., Tokyo, Japan) and R (version 3.3.0) for Windows. The grade of LDD at each disc level was analyzed using the Friedman test, followed by the Wilcoxon signed rank-sum test for multiple comparisons. Means, standard deviations (SD), and 2-sided 95 % confidence intervals (95 % CI) were calculated, and the data were expressed as the mean ± SD (95 % CI). The physical characteristics and CSAs of the athletes were compared between the LDD and non-LDD groups using an unpaired Student’s *t* test. Moreover, a paired *t* test was employed for comparisons of CSAs between the left and right sides as an asymmetrical difference. The estimation was based on an effect size of 0.5, alpha level of 0.05, and a power (1 − β) of 0.80. Statistical analysis was performed by G*power (Faul et al. 2007). The level of statistical significance was adjusted based on *p* < 0.05.

## Results

We assigned the athletes either to the LDD group (n = 69) or to the non-LDD group (n = 82) in Table 1. The prevalence of one or more LDD in combat sports athletes was 45.7 % (69/151). The physical characteristics of the combat sports athletes with and without LDD are presented in Table 1. Age, height, body weight, and BMI of the LDD group were significantly higher than those of the non-LDD group (*p* = 0.017, *p* = 0.001, *p* < 0.001, *p* < 0.001, respectively). Table 2 shows the number of graded discs at each LDD disc level in the athletes. The LDD grade was significantly different at the 5 lumbar disc levels (*p* < 0.001, Friedman test). The LDD grade at the L4–5 disc level was significantly higher than that at the L1–2, L2–3, and L3–4 disc levels (*p* < 0.001 for all, Wilcoxon signed rank-sum tests). The LDD grade at the L5–S1 disc level was also significantly higher than that at the L1–2, L2–3, and L3–4 disc levels (*p* < 0.001 for all). Thus, the LDD grade in the lower 2 lumbar discs was significantly greater than that in the other discs. On the whole, 89 (11.8 %) of 755 discs showed LDD at all lumbar intervertebral disc levels.

**Table 1:** Physical characteristics of LDD and non-LDD groups

	LDD (69 athletes)	Non-LDD (82 athletes)	*p* value
Age (years)	19.8 ± 1.1 (19.6–20.1)	19.4 ± 1.1 (19.1–19.6)	0.017
Height (cm)	173.5 ± 6.1 (172.1–175.0)	169.9 ± 6.5 (168.5–171.3)	0.001
Body weight (kg)	83.2 ± 14.8 (79.7–86.8)	73.0 ± 11.4 (70.4–75.5)	<0.001
BMI (kg/m^2^)	27.6 ± 4.1 (26.6–28.5)	25.2 ± 2.7 (24.6–25.8)	<0.001
Combat sports experience (years)	9.1 ± 4.1 (8.1–10.1)	8.4 ± 4.0 (7.5–9.3)	0.298

Data are presented as mean ± SD (95 % CI)

Combat sports experience: years of combat sports experience

*LDD* lumbar intervertebral disc degeneration, *BMI* body mass index

**Table 2:** The number of graded discs at each lumbar disc level

	Grade I	Grade II	Grade III	Grade IV	Grade V
L1–2	139 (92.1)	12 (7.9)	–	–	–
L2–3	140 (92.7)	3 (2.0)	7 (4.6)	–	1 (0.7)
L3–4	130 (86.0)	9 (6.0)	9 (6.0)	3 (2.0)	–
L4–5*	99 (65.6)	16 (10.6)	25 (16.6)	11 (7.3)	–
L5–S1*	111 (73.5)	7 (4.6)	15 (9.9)	17 (11.3)	1 (0.7)

* Significantly higher grades of lumbar intervertebral disc degeneration compared to that at L1–2, L2–3, and L3–4 disc levels (*p* < 0.001 for all). A total of 755 lumbar intervertebral discs in 151 combat sports athletes were classified into 5 grades (Grade I–V), based on Pfirrmann’s classification (Pfirrmann et al. 2001). This system uses characteristics of a disc structure, a distinction between the nucleus and annulus, MRI signal intensity, and intervertebral disc height for grading. Grade I and II denote normal. Grade III–V reflects that degeneration exists in the disc. The percentages of each grade at the disc levels are given in parentheses

The absolute CSAs of trunk muscles are available in the LDD and non-LDD groups in Table 3. The absolute CSAs of trunk muscles in the LDD group were significantly larger than those in the non-LDD group (obliques, *p* = 0.014 and total area, *p* = 0.001).

**Table 3:** Absolute cross-sectional areas (CSAs) of trunk muscles in athletes with and without LDD

CSAs of trunk muscles (mm^2^)	LDD (69 athletes)	Non-LDD (82 athletes)	*p* value
Rectus abdominis	19.6 ± 4.1 (18.6–20.6)	19.1 ± 4.1 (18.2–20.0)	0.448
Obliques	72.3 ± 13.4 (69.1–75.5)	67.8 ± 8.6 (65.9–69.7)	0.014
Psoas	30.0 ± 5.8 (28.6–31.4)	28.2 ± 6.1 (26.9–29.6)	0.082
Quadratus lumborum	20.2 ± 4.0 (19.2–21.1)	19.4 ± 4.1 (18.5–20.3)	0.224
Erector spinae plus multifidus	62.0 ± 8.8 (59.9–64.2)	59.6 ± 8.7 (57.7–61.5)	0.094
Total area	204.1 ± 23.9 (198.3–209.8)	194.1 ± 22.6 (189.2–199.1)	0.010

Data are presented as mean ± SD (95 % CI)

*LDD* lumbar intervertebral disc degeneration

Table 4 lists relative CSAs of trunk muscles to their body weight in the LDD and non-LDD groups. The relative CSAs of trunk muscles in the LDD group were significantly smaller than those in the non-LDD group (rectus abdominis, *p* = 0.011; obliques, *p* = 0.024; quadratus lumborum, *p* = 0.006; lumbar erector spinae plus multifidus, *p* = 0.002; and total area, *p* = 0.001). Totally, the LDD group had smaller relative trunk muscle CSAs than the non-LDD group. Our assessment of measurement repeatability was confirmed as a reliable agreement between two measurements for all of the CSAs (ICC 0.84–0.92).

**Table 4:** Relative cross-sectional areas (CSAs) of trunk muscles in athletes with and without LDD

CSAs of trunk muscles (mm^2^ kg^−1^)	LDD (69 athletes)	Non-LDD (82 athletes)	*p* value
Rectus abdominis	24.1 ± 5.8 (22.7–25.5)	26.6 ± 5.8 (25.3–27.8)	0.011
Obliques	88.6 ± 16.4 (84.6–92.5)	94.3 ± 14.3 (91.1–97.4)	0.024
Psoas	36.8 ± 8.4 (34.8–38.8)	39.2 ± 8.2 (37.4–41.0)	0.086
Quadratus lumborum	24.5 ± 4.7 (23.4–25.7)	26.7 ± 4.7 (25.6–27.7)	0.006
Erector spinae plus multifidus	76.0 ± 13.4 (72.8–79.3)	82.6 ± 11.6 (80.0–85.1)	0.002
Total area	250.1 ± 38.1 (240.9–259.2)	269.2 ± 32.0 (262.2–276.3)	0.001

Data are presented as mean ± SD (95 % CI). All relative CSAs are normalized by their body weight

*LDD* lumbar intervertebral disc degeneration

Table 5 indicates asymmetries of trunk muscle CSAs in the LDD and non-LDD groups. Significantly asymmetrical CSAs between the left and right sides were observed only in the LDD group (obliques: *p* = 0.040; quadratus lumborum: *p* < 0.001; and total area: *p* = 0.007). In addition, CSAs of trunk muscles on both sides in the LDD group were significantly smaller than those in the non-LDD group (rectus abdominis: left, *p* = 0.031, right, *p* = 0.011; obliques: right, *p* = 0.022; quadratus lumborum: right, *p* = 0.043; lumbar erector spinae plus multifidus: left, *p* = 0.002, right, *p* = 0.006; and total area: left, *p* = 0.005, right, *p* = 0.001).

**Table 5:** Asymmetry of cross-sectional areas (CSAs) of trunk muscles in athletes with and without LDD

CSAs of trunk muscles (mm^2^ kg^−1^)	LDD (69 athletes)	Non-LDD (82 athletes)	*p* value
Rectus abdominis			
Left	12.3 ± 3.1 (11.5–13.0)	13.3 ± 2.9 (12.7–14.0)	0.031
Right	11.8 ± 3.1 (11.8–12.6)	13.2 ± 3.1 (12.5–13.8)	0.011
*p* value	0.084	0.457	
Obliques			
Left	44.7 ± 8.7 (42.6–46.8)	47.3 ± 7.5 (45.7–48.9)	0.053
Right	43.8 ± 8.1 (41.8–45.7)	46.7 ± 7.3 (45.1–48.3)	0.022
*p* value	0.040	0.224	
Psoas			
Left	18.3 ± 4.4 (17.3–19.4)	19.4 ± 4.4 (18.4–20.3)	0.155
Right	18.5 ± 4.1 (17.5–19.5)	19.7 ± 4.0 (18.8–20.6)	0.073
*p* value	0.542	0.284	
Quadratus lumborum			
Left	23.3 ± 17.7 (19.1–27.6)	28.3 ± 19.3 (24.0–32.5)	0.105
Right	21.8 ± 17.2 (17.7–25.9)	27.9 ± 19.0 (23.7–32.1)	0.043
*p* value	<0.001	0.359	
Erector spinae plus multifidus			
Left	37.9 ± 6.5 (36.4–39.5)	41.0 ± 5.7 (39.8–42.3)	0.002
Right	38.1 ± 7.2 (36.3–39.8)	41.0 ± 6.0 (39.7–42.4)	0.006
*p* value	0.647	0.959	
Total area			
Left	125.9 ± 19.4 (121.2–130.5)	134.2 ± 16.1 (130.6–137.7)	0.005
Right	124.0 ± 19.1 (119.5–128.6)	133.7 ± 15.6 (130.3–137.1)	0.001
*p* value	0.007	0.582	

Data are presented as mean ± SD (95 % CI). All relative CSAs are normalized by their body weight

*LDD* lumbar intervertebral disc degeneration

## Discussion

The present study demonstrated that combat sports athletes with LDD in at least 1 disc from L1–2 to L5–S1 levels had a significant asymmetry of CSAs between the left and right sides in obliques, quadratus lumborum, and total area of trunk muscles. Also, athletes with LDD exhibited significant smaller relative CSAs to their body weight in the rectus abdominis, obliques, quadratus lumborum, and lumbar erector spinae plus multifidus at the L3–4 disc level parallel to the lumbar disc space, compared to those without LDD. This is the first study to have obviously shown more asymmetrical and relatively smaller trunk muscle CSAs in combat sports athletes with LDD compared to those without LDD.

Previous studies reported the association between the asymmetry of trunk muscles and lumbosacral radiculopathy. A study by Kulig et al. (2009) showed that persons who were scheduled for surgery of lumbar microdiscetomy exhibited asymmetrical CSAs of the lumbar multifidus. Hyun et al. (2007) reported that asymmetry of the multifidus CSA may reflect the denervation caused by unilateral lumbosacral radiculopathy. Similarly, decreased paraspinal muscle density was associated with spinal degeneration features at the same disc level (Kalichman et al. 2010). In an animal study, CSA of the multifidus rapidly decreased after a lumbar intervertebral disc lesion was induced experimentally. From a comprehensive standpoint, asymmetry of the muscle is observed at the location below the level of the injured disc. This phenomenon will be associated with the anatomy of the multifidus, which is innervated unisegmentally by the medial branch of the dorsal ramus. Although this study evaluated CSAs of trunk muscles only at the L3–4 disc level, multiple level evaluations can make our results more apparent. In particular, CSAs at levels below the discs with LDD may be useful to detect asymmetry of the multifidus at corresponding levels. However, we could not separate the lumbar erector spinae and multifidus into the component muscles, because these muscles did not always have clearly defined borderlines on the low filed MR device (0.3 T). Thus, a major limitation of the present study was that asymmetry of the multifidus alone could not be analyzed separately. It would be interesting to know whether the athletes with LDD have asymmetrical and relatively smaller CSAs of the multifidus compared to those without LDD. Since Table 5 shows a significantly smaller relative CSA of the lumbar erector spinae plus multifidus of the LDD group than that of the non-LDD group, we can consider that the LDD group does show significantly smaller relative CSAs of the multifidus than those in the non-LDD group.

Many previous studies have not completely analyzed trunk muscles with LDD (Gray et al. 2016; Hides et al. 2008a, b, 2010a, b; Hides and Stanton 2012; Sitilertpisan et al. 2012). Few studies of athletes have entirely examined the relationship between LDD and CSAs of trunk muscles. It is very important to assess not only the multifidus but also the other trunk muscles for appropriate monitoring of their training and physical conditions in athletes so as to prevent the incidence of LDD and LBP. Among athletes without LBP, the asymmetry and different sizes of the trunk muscles were associated with sports-specific movements and performance levels (Hides et al. 2010a, b; Hides and Stanton 2012; Iwai et al. 2008; Kubo et al. 2007; Ranson et al. 2008). Ranson et al. (2008) showed that fast bowlers in cricket had the highest asymmetry in quadrates lumborum among the lumbar muscles. They reported that fast bowlers use side flexors of the lumbar spine for frontal plane segmental stabilization to attain a position of extreme side flexion on the non-bowling arm side during the front foot contact phase. The bowling arm side quadratus lumborum is likely acting strongly to eccentrically control the non-bowling arm side and isometrically control the bowling arm side. Hence, fast bowlers show significantly larger CSAs of quadratus lumborum on the bowling arm side at multiple lumbar spine levels. Likewise, elite football players demonstrated asymmetry of trunk muscles between the side of the kicking leg and the contralateral side (Hides et al. 2010a, b). For example, the CSA of psoas was significantly greater on the kicking leg side. The asymmetry of psoas in the football players was most likely related to its function as a primary hip flexor. Here, in the combat sports athletes without LDD, no asymmetrical CSA of trunk muscles was observed between the left and right sides (shown in Table 5). Sward et al. (1990) determined symmetrical trunk muscle strength using electromyography in wrestlers. It is highly possible that combat sports athletes without LDD have symmetrical trunk muscle CSAs because they flex and rotate their trunk region equally in all directions during practice and matches. However, in this study, it is not completely clear why asymmetry of the muscles was observed only in the LDD group or why CSAs of trunk muscles in the LDD group were relatively smaller than those in the non-LDD group. Further studies may be able to discover the factors responsible for asymmetrical and relatively smaller trunk muscle CSAs in the general population and athletes with LDD.

Previous research on CSAs of trunk muscles has been conducted from various viewpoints, and one of the most studied research topics is LBP (Barker et al. 2004; Danneels et al. 2000; Demoulin et al. 2007; Keller et al. 2004). Patients with LBP seemed to exhibit asymmetrical CSAs of trunk muscles (Clark et al. 2009; Hyun et al. 2007; Kulig et al. 2009). Furthermore, LBP patients have been reported to demonstrate significantly smaller trunk muscle CSA than healthy control people. Studies among athletes indicated a similar tendency—athletes with LBP had asymmetrical and smaller CSAs of trunk muscles (Hides et al. 2008a, b, 2010a, b). However, we did not investigate the prevalence of LBP in combat sports athletes. Further studies are needed in order to identify the relationship between CSAs of the trunk muscles and LBP in combat sports athletes. To our knowledge, no previous research has compared CSAs of trunk muscles in athletes with LDD. Therefore, implementing medical check-ups including assessments of CSAs of lumbar muscles using MRI would be beneficial for elite athletes at least.

The high prevalence of LDD has been reported by previous studies in various sports (Bono 2004; Hangai et al. 2009; Koyama et al. 2013; Kulling et al. 2014; Min et al. 2010; Ong et al. 2003). Hangai et al. (2009) showed that 59.7 % of baseball players, 57.5 % of swimmers, 42.9 % of basketball players, 39.2 % of kendo competitors, 36.2 % of soccer players, and 25.6 % of runners had LDD in at least 1 disc level between L1–2 and L5–S1. In the present study, 45.7 % of the collegiate combat sports athletes (69 athletes) had LDD at one or more lumbar disc levels. Several factors contributing to LDD have been reported, such as aging, body weight, sports activities, and genetic inheritance in human beings (Ala-Kokko 2002; Battie et al. 2004; Bono 2004; Elfering et al. 2002; Liuke et al. 2005; Min et al. 2009; Parkkola and Kormano 1992). The present study found some similar factors—the LDD group was significantly older, taller, and heavier than the non-LDD group (Table 1). Older, taller, and heavier athletes need to be particularly careful in preventing LDD. Furthermore, the lower 2 lumbar discs were significantly more degenerative than the other lumbar discs in this study. Previous studies also indicated a similar trend (Bono 2004; Ong et al. 2003). A high prevalence of LDD at the 2 lower lumbar discs was also observed in a wide variety of sports athletes (Bono 2004; Hangai et al. 2009; Kaneoka et al. 2007). It was most common at the L5–S1 level and showed the most degenerative changes in a pilot study of Olympic athletes (Ong et al. 2003). LDD among athletes seems to be affected by the intensity of the sport. Of course, the lower 2 lumbar discs are structurally influenced by large loads applied to them and provided overall body support and movement in various directions. The bodies of combat sports athletes are overloaded, especially in the lumbar region, because of repeated throwing and lifting of opponents in their practice and matches.

There are three major limitations in this study. First, all relative CSAs were normalized by dividing the values by the athlete’s body weight. The method was used in the previous study (Peltonen et al. 1998), but the lean body mass should be used to eliminate the effect of fat mass. Second, MR imaging in this study was performed with weak magnetic field (0.3 T). MR devices with strong magnetic field provide detailed information and we can assess individual trunk muscles with clear borders. For example, it was reported that the CSAs of multifidus muscle were captured alone with a high resonance MRI device (Hides et al. 2008a, b; Hyun et al. 2007; Kalichman et al. 2010; Kulig et al. 2009). Lastly, only CSAs of trunk muscles at the L3–4 level were examined in this study. Multiple lumbar vertebral levels of CSAs should be examined in order to capture and estimate their more muscle volume.

## Conclusion

The present study suggests that the prevalence of LDD is associated with asymmetrical and smaller relative CSAs of the trunk muscles in collegiate male combat sports athletes. In addition, our study indicates a high prevalence (45.7 %) of LDD among combat sports athletes.
